# Treatment of non‐arthritic pseudoparetic shoulders with irreparable massive rotator cuff tears: arthroscopic procedures yield comparable midterm results to reverse arthroplasty

**DOI:** 10.1186/s12891-021-04050-w

**Published:** 2021-02-16

**Authors:** Fabian Plachel, Paul Siegert, Philipp Moroder, Leo Pauzenberger, Brenda Laky, Werner Anderl, Philipp Heuberer

**Affiliations:** 1 Center for Musculoskeletal Surgery, Charité - Universitaetsmedizin, Berlin, Germany; 2 Department of Orthopedics and Traumatology, Paracelsus Medical University, Salzburg, Austria; 3 Department of Orthopedics, St. Vincent Hospital Vienna, Hartmanngasse 15/9, 1050 Vienna, Austria; 4 Austrian Research Group for Regenerative and Orthopedic Medicine, Hartmanngasse 15/9, 1050 Vienna, Austria

**Keywords:** Irreparable massive rotator cuff tear, Pseudoparesis, Reverse total shoulder arthroplasty, Arthroscopic treatment, Midterm results

## Abstract

**Background:**

Irreparable massive rotator cuff tears (IMRCTs) are a well-known cause for functional limitation and difficult to treat. Although several joint-preserving as well as joint-replacing procedures were found to provide pain relief and gain of function, midterm results are scarce, particularly in pseudoparetic shoulder joints unaccompanied by severe osteoarthritis. The purpose of this study was to compare the midterm functional outcomes of arthroscopic procedures to those of reverse total shoulder arthroplasty (RTSA) in pseudoparetic shoulders with IMRCTs unaccompanied by severe osteoarthritis.

**Methods:**

All patients who underwent either joint-preserving (group A) or joint-replacing (group B) procedures for IMRCT unaccompanied by severe osteoarthritis with a pseudoparetic shoulder function were retrospectively included. Clinical assessment included the Constant Score (CS), the Subjective Shoulder Value (SSV) and the Visual Analog Score (VAS) at baseline and at latest follow-up. Furthermore, the complication and revision rates were assessed.

**Results:**

Overall, a total 56 patients were included of whom each 28 patients formed group A (male, 36%) and B (male, 53%) with a mean patient age at time of surgery of 70 ± 7 years and 72 ± 7 years, respectively. The mean follow-up period was 56 ± 17 months. At final follow-up, the total CS (group A: 66 ± 14 points; group B 54 ± 15 points) was significantly increased after arthroscopic treatment when compared to RTSA (*p*=0.011). However, no significant differences were detected with SSV (*p*=0.583) and VAS (*p*=0.536). Although complication rate (11% versus 18%) was not significantly different (*p*=0.705), number of revision surgeries was significantly higher in group B when compared to group A (*p*=0.041).

**Conclusions:**

In non-arthritic pseudoparetic shoulders, both joint-preserving and joint-replacing procedures yielded good clinical midterm outcomes for the treatment of degenerative IMRCTs. Despite of comparable functional and satisfactory functional improvement, increased complication rates and surgical invasiveness outweigh the benefits of primary RTSA and therefore reserve this procedure to a second-line treatment in pseudoparetic patients without any signs of severe cuff arthropathy.

## Introduction

The term massive rotator cuff tear (RCT) refers to a demanding shoulder disorder defined by a complete detachment of at least two tendons of the rotator cuff [1]. Over time, massive RCTs lead to chronic rotator cuff degeneration with accompanied myotendinous retraction, muscle atrophy, and fatty infiltration [2–5]. Furthermore, additive disruption of the superior capsule causes cranial migration of the humeral head with consecutive osteoarthritis [4, 6]. Patients with massive RCTs generally present with complaints of pain and weakness [7, 8]. Larger tears increase the likelihood of functional loss or even pseudoparesis [9], which is defined as an active shoulder elevation of less than 90° despite of a free passive range of motion [10].

Attempts of anatomical reconstruction often result in pain relief and improved function, but the ability to achieve watertight repair is poor and reported failure rates are higher than 50 % [11]. Determining what constitutes an irreparable massive RCT (IMRCT) is difficult, as multiple factors must be considered, including tear-specific characteristics along with patient-related factors and surgeon’s ability. Indeed, appropriate management of patients with IMRCTs without glenohumeral osteoarthritis remains a challenge. A number of palliative treatment options are available, from non-operative to simple debridement with or without biceps tenotomy, open as well as arthroscopic partial repair, patch augmentation, superior capsule reconstruction, tendon transfer, and reversed total shoulder arthroplasty (RTSA) [10, 12–15]. Given the potential of unfavorable results with arthroscopic debridement [16], it is widely accepted to partially repair the rotator cuff most feasibly to convert an unbalanced tear to a functional RCT by obtaining a balanced force-couple [17]. Besides that, RTSA provides a promising option for elderly patients with massive RCTs that may otherwise be considered irreparable or at significant risk of failure, particularly those with less than 90° of abduction [18, 19].

As current literature lacks in comparing different options to treat IMRCTs without advanced osteoarthritis, the optimal treatment for patients suffering concomitant pseudoparesis is yet unknown. The purpose of this retrospective study was to determinate the midterm functional results in patients with pseudoparetic shoulders caused by IMRCT following arthroscopic joint-preserving procedures (including debridement and partial repair) and RTSA. We hypothesized that there is no significant difference in midterm results after arthroscopic treatment and RTSA.

## Materials and Methods

For the purpose of this retrospective study, all consecutive patients who underwent either arthroscopic joint-preserving or open joint-replacing procedures for the treatment of IMRCTs between 2006 and 2009 were assessed for eligibility.

Of whom, we included all patients treated with arthroscopic debridement or partial repair (group A) and those who received RTSA (Anatomical Shoulder Inverse/Reverse System, Zimmer, Warsaw, USA) (group B). Further inclusion criteria for both treatment options were: (I) IMRCT involving at least three tendons with fatty infiltration of the supraspinatus (SSP) or infraspinatus (ISP) muscles greater than stage 2 according to Goutallier et al. [5] or Fuchs et al. [20], detected by preoperative CT or MRI slice-imaging, respectively; (II) absence of discernible osteoarthritis on preoperative radiographs defined as stage 1 to 3 using the Hamada classification system (stage 1 defined as an Acromiohumeral Interval (AHI) ≥ 6mm, stage 2 with AHI ≤ 5mm, stage 3 with AHI ≤ 5mm and acetabularization of coracoacromial arch, stage 4 with additional glenohumeral narrowing without acetabularization (4a) and with acetabularization (4b), stage 5 with additional humeral head necrosis [21]); (III) pseudoparetic shoulder function (active elevation < 90° in the presence of free passive range of motion) evaluated clinically as formerly described by Tokish and colleagues [9]; (IV) failed conservative treatment for at least 6 months with persistent debilitating shoulder pain and severely impaired function; and (V) minimum follow-up of 36 months.

**Fig. 1:**
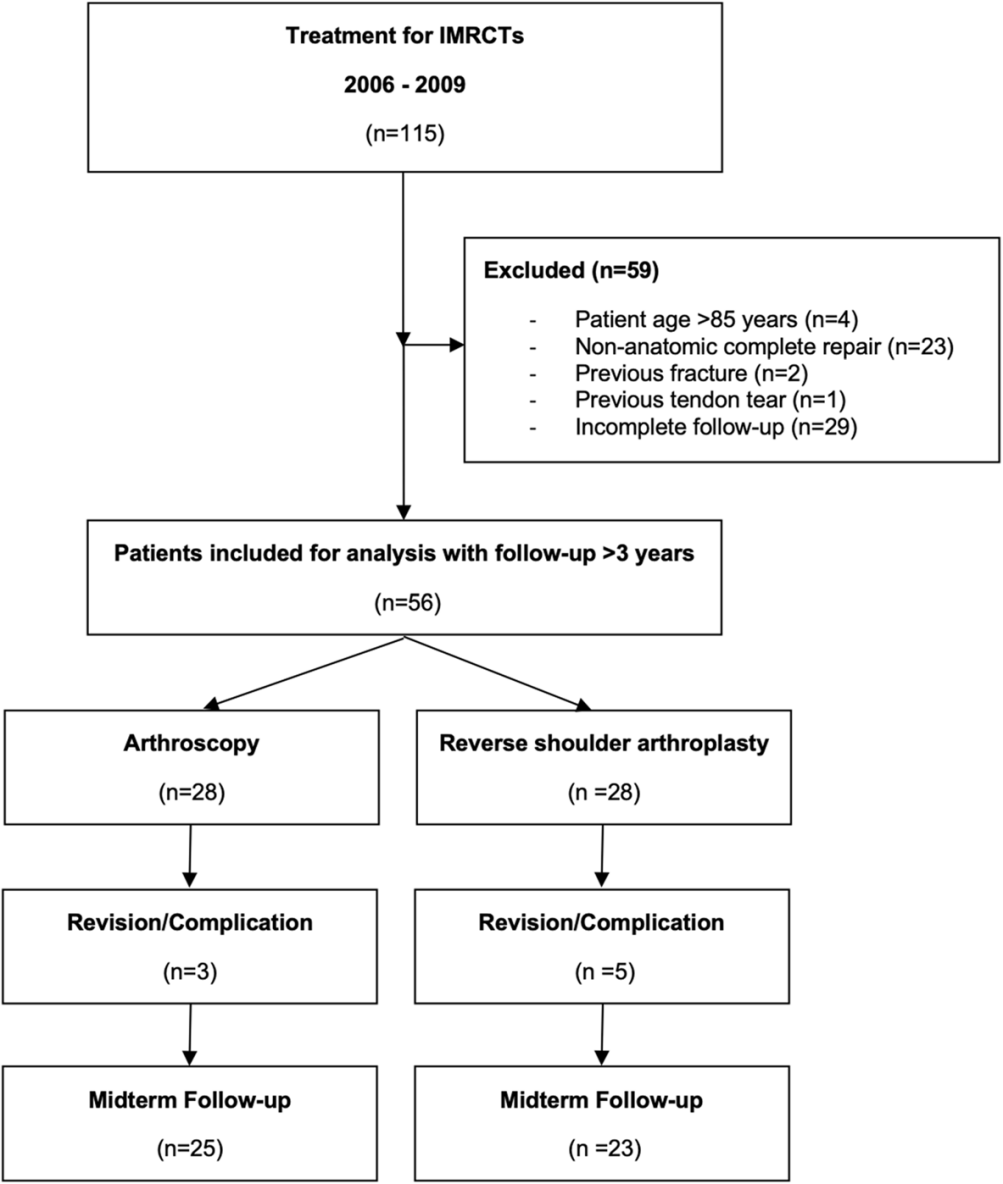
Flowchart of the enrollment and analysis. IMRCT = irreparable massive rotator cuff tear

We excluded all patients older than 85 years at time of surgery, those in whom a complete non-anatomical (i.e. medialized) arthroscopic repair of the tendons was successfully performed, those with previous fractures or tendon tears other than the rotator cuff and the long head of the biceps (LHB) tendon around the affected shoulder joint and those with an incomplete follow-up. The flow chart given in Fig. 1 illustrates the patient selection.

Prior to surgery, each patient received preoperative x-ray examination in three standard planes and slice imaging including either computed tomography or magnetic resonance imaging. For the purpose of this study and to minimize observation bias, two independent investigators retrospectively performed all radiographic gradings, working in consensus. First, plain radiographic imaging were assessed for cuff arthropathy of the affected shoulder and graded using the Hamada classification [21]. Further, tendon involvement was assessed by slice imaging preoperatively and was confirmed intraoperatively. The global fatty degeneration index (GFDI) was calculated as previously described by Goutallier and colleagues [22].

 In general, indication for surgery was made individually in accordance with the patient´s preference and performed after providing informed consent. All procedures were performed at a single institution by three experienced shoulder surgeons. In group A, arthroscopic treatment was performed in a standard fashion [11]. The patient was placed in a lateral decubitus position with the affected arm prepared and draped. After diagnostic arthroscopy, the rotator cuff was carefully debrided and mobilized to assess reparability. If the horizontal force-couple was considered reparable, vertical mattress sutures using a double-loaded suture anchor were accomplished for the ISP and the subscapularis (SSC) tendon. Beyond that, subacromial soft tissue decompression, while preserving the coracoacromial ligament, was performed in 90 % of the cases together with a tenotomy of the LHB tendon in 57 %. The LHB tendon was already missing in 36 % of the patients. The surgical technique for implantation of the RTSA was performed in modification to the method previously described by Werner et al. in 2005 [10]. Duration times of the surgical procedures were recorded. Patients were prospectively followed through their standard of care patient appointments and all midterm data were then retrospectively reviewed in 2019 for study purpose. The institutional review board approved this retrospective study.

Overall, group A consisted of 28 patients (15 male, 13 female) with a mean age of 70 ± 7 (range, from 59 to 81 years) at time of surgery. Of whom, 13 patients (46 %) were treated by debridement and 15 patients (54 %) by partial repair of the rotator cuff.

**Fig. 2:**
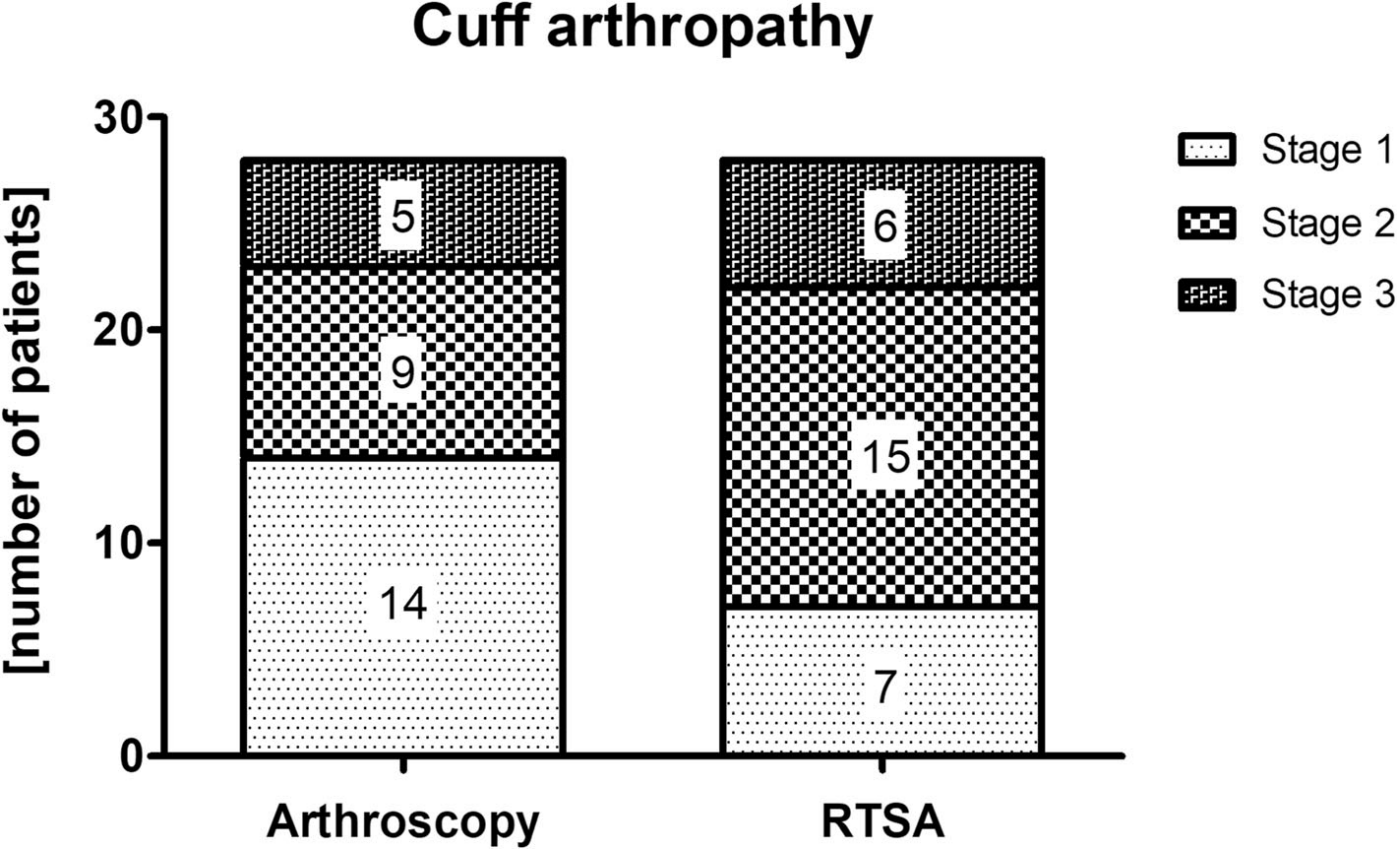
Distribution of cuff arthropathy for both groups

Group B also included 28 patients (10 male, 18 female) with a mean patient age of 72 ± 7 years (range, 56 to 85 years) at time of surgery. Severity of cuff arthropathy was comparable between both group A and B (p = 0.141) (Fig. 2). Further baseline data are summarized in Table 1.

**Table 1:** Baseline patient characteristics (group A = arthroscopic treatment; group B = reverse total shoulder arthroplasty)

Variables	Group A *N* = 28	Group B *N* = 28	*p-value*
Age at time of surgery, years^a^	70 ± 7	72 ± 7	*0.228*
Gender, male, %	36	53	*0.179*
Dominant arm, %	82	77	*0.670*
Previous surgery, %	0	35	*0.001*
Tendon involvement^b^, %			
SSP:	100	100	
full-tendon	100	100	*> 0.999*
partial-tendon	0	0	*> 0.999*
ISP:	100	100	
full-tendon	50	71	*0.450*
partial-tendon	50	39	*0.233*
SSC:	100	100	
full-tendon	21	39	*0.122*
Partial-tendon	79	61	*0.341*
Global fatty degeneration index^a^	1.7 ± 0.7	1.9 ± 0.3	*0.559*

^a^Data are reported as mean ± SD, ^b^full-thickness tendon tear

### Clinical Assessment

Each patient was pre- and postoperatively evaluated by two independent orthopedic specialists using the same clinical techniques. The Constant Score (CS) as primary outcome measurement together with the Subjective Shoulder Value (SSV, %), Visual Analog Scores (VAS) and comprehensive physical evaluation including active abduction, forward flexion, external and internal rotation was evaluated at baseline and at latest follow-up. Improvement in outcome measures from pre- to postoperatively were then presented as delta-values (Δ). In addition, each patient was routinely asked to provide their satisfaction with surgery (very satisfied, satisfied, moderate satisfied, not satisfied) [23, 24]. All surgical complications and required revision surgeries were documented.

### Statistical analysis

Statistical analyses were performed with IBM SPSS Statistics 24.0 software (IBM, Armonk, NY, USA) with the p-values being 2-tailed and the alpha level set to 0.05. According to a previous study comparing short-term clinical results after arthroscopic debridement and partial repair for IMRCTs [25], a power analysis showed that the minimum sample size was 13 to find significant differences in CS with statistical power of 0.95 (α = 5 %). Based on an assumed 20 % dropout rate, 16 patients were needed in each group. Furthermore, descriptive statistics (means, standard deviation, minimum and maximum values of continuous variables) were calculated. To analyze statistical differences between group A and B as well as between pre- and postoperative measures, either the independent t test or the Mann-Whitney U test as well as the paired t test or the Wilcoxon matched-pair test (depending on variable distribution) was conducted.

## Results

**Fig. 3:**
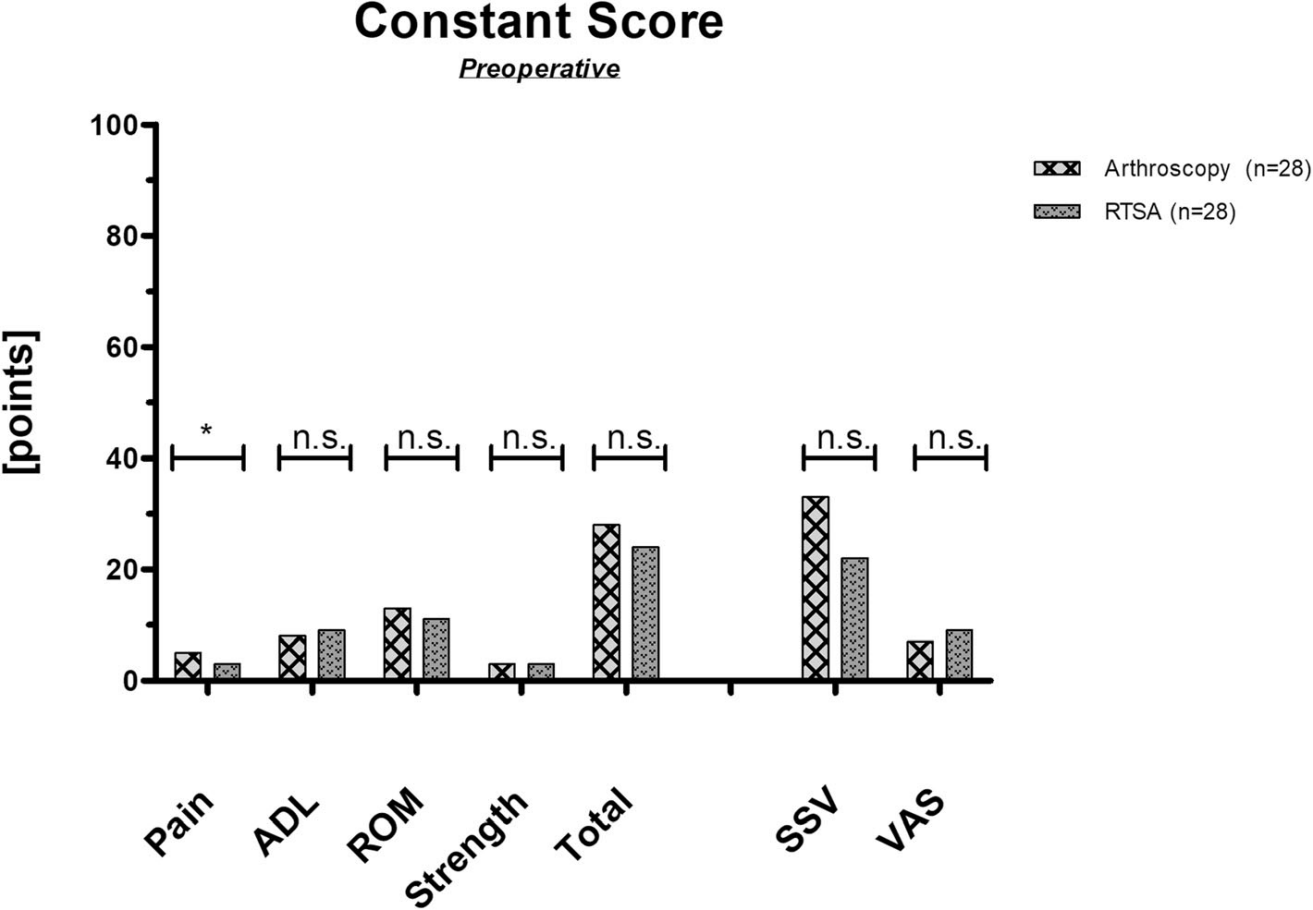
Preoperative outcome measures for group A (arthroscopic treatment) and group B (reverse total shoulder arthroplasty (RTSA)). Significance level indicated (*). ADL = activity of daily life; ROM = range of motion; SSV = subjective shoulder value; VAS = visual analogue scale

Overall, all preoperative clinical scores, except for pain, were comparable between group A and group B (Fig. 3). Among group B, previous surgery was not associated with inferior total CS (*p* = 0.566) and SSV (*p* = 0.692). Active range of motion did not differ between both groups with regard to shoulder abduction (group A: 78° 18, group B: 68° ± 18; *p* = 0.236) and forward flexion (group A: 88° ± 32, group B: 78° ± 37; *p* = 0.145).

During the follow-up period, 3 patients from group A (11 %) were revised due to a superficial infection (n = 2) or anchor loosening (n = 1). In group B, 5 patients (18 %) sustained a total of 8 complications and underwent a total of 6 revision surgeries. Further information is summarized in Table 2.

**Table 2:** Required revision surgeries on the affected shoulder during follow-up period

	Technique	Age^a^	Gender	Previous surgery	Time period^b^	Reason	Revision surgery	f/u^c^	Satisfaction^d^	Δ SSV	Δ CS
**Group A**	PR	68	Male	No	9 days	Superficial infection	Arthroscopic joint lavage	4	Satisfied	60	46
	PR	73	Male	No	9 days	Superficial infection	Open joint lavage	5	Satisfied	30	30
	PR	68	Female	No	40 days	Anchor loosening	Arthroscopic anchor removal	3	Satisfied	80	52
**Group B**	RTSA	85	Male	No	78 days	Early dislocation	Closed reduction				
					140 days	Second dislocation	Humeral revision	1	n.a.	n.a.	30
	RTSA	73	Male	No	28 days	Early dislocation	Closed reduction				
					6 years	Static subluxation	Glenoid revision	6	Satisfied	20	18
	RTSA	77	Female	Yes	2.5 years	Acromion stress fracture	Osteosynthesis	3	n.a	n.a	14
	RTSA	79	Female	No	1 year	Aseptic glenoid loosening	Glenoid revision				
					5 years	Aseptic glenoid loosening	Conversion to HA	6	n.a.	n.a.	9
	RTSA	69	Female	No	3 years	Traumatic axillary nerve palsy	Open patch plastic	4	n.a.	n.a.	48

^a^patient age at time of initial surgery; ^b^time period between initial surgery and revision surgery; ^c^time period between initial surgery and latest follow-up (f/u); ^d^satisfaction with initial treatment; *SSV* Subjective Shoulder Value; *CS *Constant Score; Δ- value = difference between pre- and postoperative score; *PR *partial repair; *RTSA *reverse total shoulder arthroplasty; *n.a.* not available

Although complication rates were not significantly different (*p* = 0.705), the number of revision surgeries was significantly higher in group B when compared to group A (*p* = 0.041). These patients were excluded from further analysis.

**Fig. 4:**
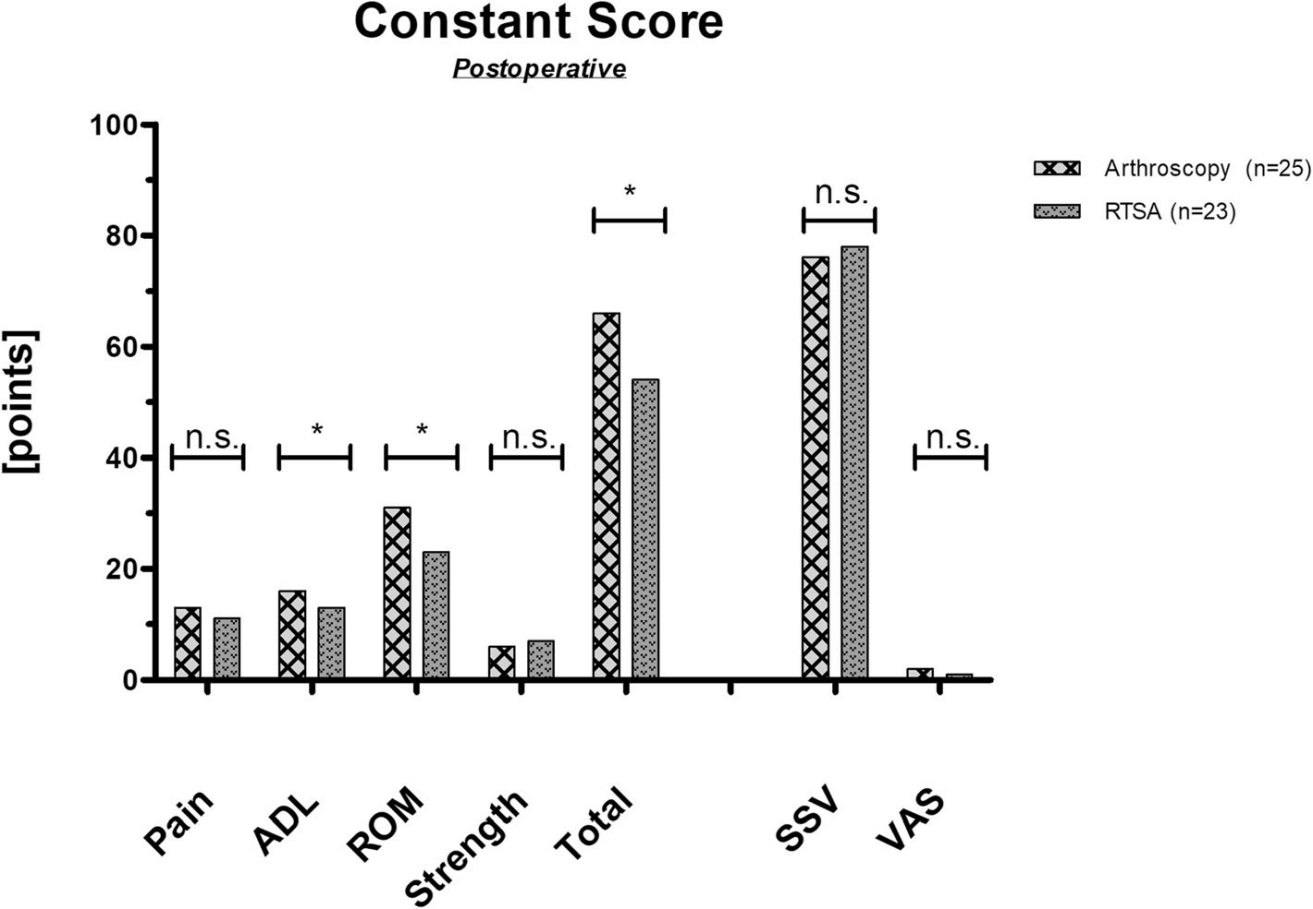
Postoperative outcome measures. Significance level indicated (*). RTSA = reverse total shoulder arthroplasty; ADL = activity of daily life; ROM = range of motion; SSV = subjective shoulder value; VAS = visual analogue scale

The mean follow-up period was 56 ± 17 months (range, from 36 to 93 months) with a significant difference between group A (44 ± 9 months) and group B (68 ± 14 months) (*p* < 0.05). The mean duration of surgery was significantly higher (*p* < 0.05) in RTSA when compared to arthroscopic procedures with a mean of 95 ± 20 minutes (range, from 63 to 136 minutes) and 54 ± 35 minutes (range, from 12 to 156 minutes), respectively. The majority of patients in both groups were satisfied with their procedure (group A: 84 %; group B: 87 %; p = 0.755). The total CS was significantly better after arthroscopic treatment (66 ± 14) when compared to RTSA (54 ± 15) (*p* = 0.011) (Fig. 4). No significant differences were detected with SSV (*p* = 0.583) and VAS (p = 0.536).

Improvement of total CS from pre- to postoperatively was significant in both group A (mean ∆ 37 ± 13 points; p = 0.001) and group B (mean ∆ 31 ± 21 points; p = 0.001). Further information on score improvement is found in Table 3.

**Table 3:** Functional improvement from pre- to postoperatively (∆-value)

Variables^a^	Group A *N* = 25	Group B *N* = 23	*p-value*
**Constant Score, points**			
∆-value	37 ± 13	31 ± 21	*0.224*
**Subjective Shoulder Value, %**			
∆-value	43 ± 18	62 ± 15	*0.001*
**Visual Analog Scale, points**			
∆-value	5 ± 3	7 ± 2	*0.089*

^a^Data are reported as mean ± SD

With regard to the specific arthroscopic treatment, we did not find any significant differences between debridement (*n* = 13) and partial repair (*n* = 12) at final follow-up. Neither total CS (64 ± 18 points vs. 68 ± 9 points; *p* = 0.586) nor SSV (76 % ± 11 versus 76 % ± 16; *p* = 0.0955) as well as VAS (2 ± 2 points vs. 1 ± 2 points; *p* = 0.758) were influenced by final treatment. Furthermore, score improvement was comparable (CS: 39 ± 12 points vs. 35 ± 15 points, *p* = 0.468; SSV: 42 % ± 13 vs. 43 % ± 24, *p* = 0.853; VAS: 5 ± 2 points vs. 5 ± 3 points, *p* = 0.877).

## Discussion

The most important finding of this study is that in non-arthritic shoulders both joint-preserving and joint-replacing procedures yielded good clinical midterm outcomes for the treatment of degenerative IMRCTs.

A variety of factors need to be considered for treatment of massive RCTs. Despite of modern preoperative imaging techniques, repairability of massive RCTs cannot always be predicted. Indeed, it was previously shown that a complete repair is most favorable in terms of functional and satisfactory improvement, with rates as low as 2.4 % needing a secondary conversion to RTSA in a long-term follow-up [26]. However, Goutallier et al. stated that a fatty muscle infiltration of more than 50 % resulted in inferior functional outcome following complete rotator cuff repair [5]. On the contrary, Burkhart et al. found that even patients with muscle degeneration above 50 % would significantly benefit from an arthroscopic reattachment or partial repair [13]. These findings were supported by Cuff et al. investigating arthroscopic partial repair for IMRCTs [27]. Although patients without osteoarthritis were evaluated in their study, in contrast to our population, pseudoparetic patients were excluded. Our data supports that even these patients would benefit from a partial repair or even simple debridement. Considering found satisfaction, pain relief, and functional gain, primary arthroscopic treatment could be considered in the management of selected patients with IMRCTs unaccompanied by glenohumeral osteoarthritic changes. Imperatively, treatment options should be discussed thoroughly with the patient. Even conservative treatment was shown to yield satisfactory results in patients with lower demands and successful compensation upon midterm follow-up, however, progression of glenohumeral osteoarthritis as well as possible conversion from repairable to irreparable cuff tears were observed [4]. After 10 years, Zumstein et al. reported osteoarthritic changes in 61 % of patients with a significant progression between mid- and long-term assessment following open rotator cuff repair for massive RCTs [28]. A recent long-term follow-up after open rotator cuff repair by Herve et al. reported secondary glenohumeral osteoarthritis in 29 % of all patients with even higher rates in patients with massive RCTs [29]. Both of these studies excluded irreparable lesions in their evaluation. Although secondary glenohumeral osteoarthritis following incomplete rotator cuff repair was not evaluated in our study, this progressive condition might be an important mid- to long-term issue with possibly even higher rates than reported with full repair. In the context of the patient’s age, the decision to undertake first-line arthroscopic treatment should consider these secondary consequences. Denard et al. investigated the effectiveness of arthroscopic complete and partial repair for massive RCTs with active forward flexion and elevation under 90° and found a success rate for reversing the pseudoparetic state in 94.6 % of all patients [30]. In addition to their findings our study shows that the results after arthroscopic treatment are comparable in functional outcome to those following RTSA.

Sirveaux at al. propose RTSA to be reserved for elderly patients in consideration of concomitant complications, such as material loosening, notching and infection, as well as limited revision options [31]. Zumstein et al. reported an overall complication rate after RTSA of 24 %, with instability and infection being most noticeable [32]. Our study underlines those high complication rates, with instability being the major reason for revision surgery (Table 3). Long-term results of RTSA in more active patients before the age of 60 years showed persistent improvement in range of motion and pain relief, but also reported substantial complication rates [33]. Recent cost-effectiveness analyses found primary arthroscopic procedures even upon potential failure with secondary conversion to RTSA to be the economically superior strategy [34, 35]. Boileau et al. examined patients treated with RTSA after failed rotator cuff repair. They found overall significant improvement in range of motion and pain relief, although results seem to be inferior to primary RTSA [36]. Underlining these findings, a recent study by Carducci et al. investigated influences on inferior functional outcomes following RTSA without further complications and found a significant correlation to prior failed surgical interventions [37]. In our study population we did not observe any differences in shoulder function within the RTSA group. However, the smaller patient number could lead to biased results.

Our study indicates that despite of comparable functional and satisfactory improvement, increased complication rates and surgical invasiveness outweigh the benefits of primary RTSA and therefore reserve this procedure to a second-line treatment in pseudoparetic patients without any signs of severe cuff arthropathy. Nevertheless, certain limitations have to be considered. First, all inherent disadvantages of retrospective evaluations apply to the present study. Due to the small sample size, the study might be underpowered to detect small effect sizes also within the groups. In order to counteract the limited cohort sizes and underline homogeneity for better comparability, we strictly defined inclusion and exclusion criteria for comparison and conducted a power analysis prior to the investigation. Although an individualized treatment was sought for each patient, it cannot be ruled out that a certain selection bias can occur, primarily due to the patient’s activity levels. Another potential bias is the difference in follow-up time. Mean follow-up for group A was shorter, which could underestimate a decline in function and therefore patient´s satisfaction over time. All procedures were performed by three different surgeons, which could possibly bias the outcome. Given the level of experience and years of training in the same institution this is highly unlikely.

As treatment in patients with severe shoulder complaints secondary to IMRCTs is still a great challenge and literature lacks in comparison studies at the highest level, it is not possible to give an algorithmic approach. Nevertheless, we were able to show that arthroscopic treatment of patients with massive RCTs showed comparable results to those of RTSA, even if complete repair was not achieved. Thus, our study is of great value to further simplify decision-making. Given the good clinical results, arthroscopic treatment is highly efficient in the treatment of IMRCTs without osteoarthritis, even in pseudoparetic shoulder. However, possible implications, such as secondary glenohumeral osteoarthritis, potentially inferior functional outcome following secondary joint replacement, and economic factors need to be clarified in further research.

## Conclusions

Both, arthroscopic procedures and RTSA in the treatment of IMRCTs achieved considerable midterm improvement in pain relief and gain of function in pseudoparetic shoulders without glenohumeral osteoarthritis.

## Data Availability

The datasets used and analysed during the current study are available from the corresponding author on reasonable request.

## Abbreviations

RCT: Rotator cuff tear
IMRCT: Irreparable massive rotator cuff tears
RTSA: Reverse total shoulder arthroplasty
SSP: Supraspinatus
ISP: Infraspinatus
SSC: Subscapularis
AHI: Acromiohumeral interval
LBS: Long head of the biceps
GFDI: Global fatty degeneration index
CS: Constant Score
SSV: Subjective Shoulder Value
VAS: Visual analogue scale
